# Patternable PEDOT nanofilms with grid electrodes for transparent electrochromic devices targeting thermal camouflage

**DOI:** 10.1186/s40580-015-0051-9

**Published:** 2015-10-01

**Authors:** Bumsoo Kim, Jong Kwan Koh, Junyong Park, Changui Ahn, Joonmo Ahn, Jong Hak Kim, Seokwoo Jeon

**Affiliations:** 1 Department of Materials Science and Engineering, Korea Advanced Institute of Science and Technology (KAIST), 335 Gwahangno, 305-701 Yuseong-gu, Daejeon Republic of Korea; 2 KAIST Institute for the Nanocentury, Graphene Research Center, Korea Advanced Institute of Science and Technology (KAIST), 335 Gwahangno, 305-701 Yuseong-gu, Daejeon Republic of Korea; 3 Department of Chemical and Biomolecular Engineering, Yonsei University, 262 Seongsanno, 120-746 Seodaemum-gu, Seoul Republic of Korea; 4 Agency for Defense Development (ADD), 305-152 Yuseong-gu, Daejeon Republic of Korea

## Abstract

This paper reports a new type of transmitting mode electrochromic device that uses the high-contrast electrochromism of poly(3,4-ethylenedioxythiophene) (PEDOT) and operates at long-wavelength infrared (8–12 μm) . To maximize the transmittance contrast and transmittance contrast ratio of the device for thermal camouflage, we control the thickness of the thin PEDOT layer from 25 nm to 400 nm and develop a design of grid-type counter electrodes. The cyclability can be greatly improved by selective deposition of the PEDOT film on grid electrodes as an ion storage layer without any loss of overall transmittance. The device with optimized architectures shows a high transmittance contrast ratio of 83 % at a wavelength of 10 μm with a response rate under 1.4 s when alternating voltage is applied. Captured images of an LED lamp behind the device prove the possibility of active, film-type camouflage against thermal detection.

**Electronic supplementary material:**

The online version of this article (doi:10.1186/s40580-015-0051-9) contains supplementary material, which is available to authorized users.

## Background

Electrochromism is defined as a reversible color change of materials due to applied external voltage [1]. The insertion or extraction of ions using electrochemical potentials changes the electronic states of the electrochromic materials; such a process can be used for the fine tuning of absorbance, transmittance, and reflectance. Most electrochromic devices operating in the visible and near-infrared wavelengths have been used for optical displays [2, 3], smart windows [4, 5], sunglasses [6], and auto-dimming rear view mirrors due to the advantages of low-power consumption [7], reversible color change [8], and image fixation without a continuous supply of electricity [9]. Although less well known, electrochromic materials have electrochromism in the range of mid-wavelength infrared (MWIR) [10] to long-wavelength infrared (LWIR) [11] good enough to be useful in military devices for IR detection/camouflage. For example, the rapid development of forward-looking IR systems has led to a demand for efficient IR electrochromic devices for military camouflage [12]. Moreover, the importance of IR electrochromic devices is increasing due to the strong interest in satellite thermal control [13, 14].

Among various electrochromic materials, transition metal oxides, particularly tungsten oxides, are the most studied materials for IR electrochromism [15]. Franke et al. reported electrochromic devices based on tungsten oxides that had thermal emittance modulation of ~20 % [16]. Bessiere et al. reported an IR emissivity modulator made of monohydrated tungsten oxide with 30 % reflectance contrast [17]. Still, the low modulation of the thermal emittance of metal oxides, in addition to the difficulty in processing and low flexibility due to brittle fracture, requires organic materials for a flexible or wearable system for special applications. Recently, conducting polymers have received great attention due to their advantages of light weight, flexibility, easy deposition, and fast switching times [18–21]. Some valuable studies have explored electrochromic devices based on conducting polymers that can modulate IR emissivity or reflectance [22, 23]. The specular reflectance of an electrochromic device can be adjusted between 0.2 and 0.65 at 12 μm by using polyaniline (PANI) with camphor sulfonic acid (CSA) [24]. Emissivity of an electrochromic device varying from 0.32 to 0.79 was obtained using a unique combination of PANI/poly(diphenyl amine) and dopant matrix [25]. More recently, a copolymer of aniline and o-anisidine was studied, and the different average emissivity dynamic of the film was 0.408 in the wavelength of 8 – 14 μm regions [26]. However, to the best of our knowledge, there have been few studies of electrochromic devices that have demonstrated control of MWIR transmittance [27] and none that have demonstrated control of LWIR transmittance.

In this paper, we present the design and characterization of electrochromic devices that use poly(3,4-ethylenedioxythiophene) (PEDOT) thin film as a working electrode to modulate the transmittance of LWIR light in the range of 7.5 to 13 μm. Transmittance of neutral and doped states of the PEDOT film decrease with increasing the film thickness; we varied the film thickness to optimize between the transmittance contrast (T_neutral_-T_doped_) and the transmittance contrast ratio ((T_neutral_-T_doped_)/T_neutral_). We adopted grid-patterned gold (unlike indium tin oxide (ITO), which is highly reflective (>80 %) in the LWIR spectral region due to the free carrier concentration) to make a transparent electrode in LWIR, while still maintaining low sheet resistance (31.7 Ω/□) for the stable operation of the device. Moreover, for stable cyclability without gold degradation while maintaining transparency, grid-patterned PEDOT, deposited by selective deposition of electrochemical polymerization, was used as a counter electrode. We measured the transmittance of the device under a variation of the potentials and took thermal images using an infrared camera. The device showed a transmittance contrast ration of 83 % in 1.4 s. There was good correlation between the transmittance data and the thermal images. The results have broad implications for polymeric device design for LWIR applications, such as IR camouflage and thermal energy control for smart windows and satellites.

## Methods

### Materials

A germanium window with AR coating purchased from IRKen was used as an LWIR transparent substrate. Double-side polished (DSP) silicon wafers purchased from iTASCO were used as an IR transparent substrate. Acetonitrile, propylene carbonate, pyridine, 3,4-ethylenedioxythiophene (EDOT), and lithium perchlorate were purchased from Aldrich. Acetone and isopropyl alcohol were purchased from Samchun. Iron(III) p-toluenesulfonatehexahydrate (Fe tosylate) and 1-butanol were purchased from Aldrich.

### Preparation of grid-patterned gold/substrate

The substrate was cleaned with acetone, isopropyl alcohol, and deionized water by sonication for 5 min. Photoresist (AZ5214, MicroChem, 2000 rpm for 30 s) was spun-cast onto the substrate and then grid-patterned with linewidths of 5 and 20 μm and line spaces of 200 and 500 μm. On the grid-patterned substrate, 3 nm of chromium film and 30 nm of gold film were deposited by e-beam evaporation.

### Fabrication of working and counter electrodes

**Scheme 1:**
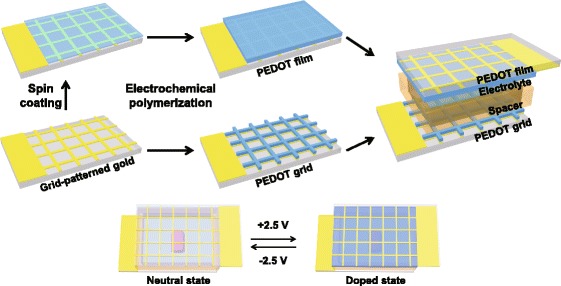
Schematic representation of the transmissive infrared electrochromic device

A working electrode of PEDOT film was fabricated by two steps as shown in Scheme 1. First, a PEDOT thin film (20 nm) was formed by solution polymerization, and then PEDOT was additionally synthesized by electrochemical polymerization. To make the PEDOT thin film, an EDOT monomer (0.20 g), pyridine retardant (0.306 g), Fe tosylate oxidant (2.7 g) and 1-butanol solvent (10.0 g) were thoroughly mixed for a few minutes, and the solution was filtered to remove impurities or polymerized PEDOT. After that, it was spin coated onto the grid-patterned gold/substrate (2000 rpm, 30 s), which was followed by polymerization at 80 °C for 5 min on a hot plate. After polymerization, the PEDOT thin film was washed with isopropyl alcohol to remove the unreacted oxidant. Then, the film was dried using nitrogen gas.

After the PEDOT thin film was formed, additional PEDOT was deposited by electrochemical polymerization. The VersaSTAT3-200 (Princeton) potentiostat/galvanostat was applied to perform all electrochemical polymerization in this work. A conventional three-electrode system was used in this work and it consisted of a reference electrode (Ag/AgCl (KCl_sat_)), a working electrode, and a counter electrode (platinum mesh). PEDOT was polymerized from a 0.1 M LiClO_4_ acetonitrile solution with an EDOT concentration of 0.01 M under galvanostatic condition. The current density was fixed at 0.3 mA/cm^2^, and the deposition time was varied for different deposition amounts.

A counter electrode of grid-patterned PEDOT was fabricated by a single step. Grid-patterned PEDOT was electrochemically polymerized on the grid-patterned gold/substrate. Without any spin-coated PEDOT film, electrochemical polymerization of PEDOT occurred only on the gold-deposited area which was conductive, not on the uncoated area which was insulated. The same conditions of electrochemical polymerization were applied except for the current density and the deposition time to control the thickness of the grid-patterned PEDOT. The current density was reduced because the area where synthesis occurred was smaller than the area of the substrate.

### Preparation of electrochromic devices

As shown in Scheme 1, the device was composed of a substrate/grid-patterned gold/PEDOT film/electrolyte with spacer/grid-patterned PEDOT/grid-patterned gold/substrate. The device was assembled with the PEDOT film as a working electrode, grid-patterned PEDOT as a counter electrode, and 1.0 M LiClO_4_ in propylene carbonate as an electrolyte, with a spacer between the grid-patterned gold/AR-coated germanium. The thickness of the electrolyte was controlled by the thickness of the spacer; this spacer could be either double-sided tape or SU-8 in a size range from 2 to 250 μm.

### Characterization methods

The thicknesses of the PEDOT films were measured with an atomic force microscope (AFM, N8 NEOS SENTERRA system from Bruker). The IR transmittances of the PEDOT films were measured by Fourier transform infrared spectroscopy (FT-IR, IFS66V/S & HYPERION 3000 from Bruker). Thermal images of the device were captured using an FLIR T400, which has a spectral range of 7.5 to 13 μm.

## Results and discussion

**Fig. 1:**
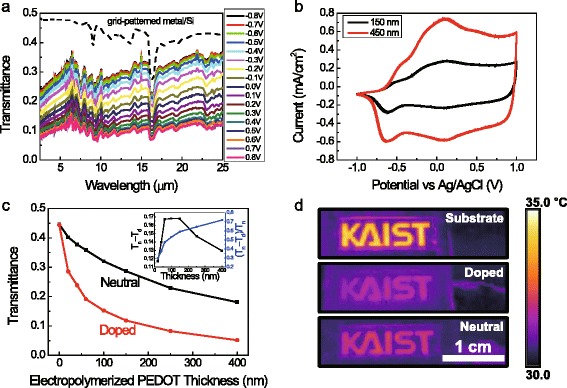
**a** Transmittance spectra from 2.5 μm to 25 μm of 150 nm PEDOT film deposited on grid-patterned gold/DSP silicon obtained at every 0.1 V between +0.8 V and **−**0.8 V versus Ag/AgCl(KCl_sat_) reference electrode. **b** Cyclic voltammogram of PEDOT films from −1.0 V to 1.0 V (versus Ag/AgCl(KCl_sat_), at a scan rate of 50 mV/s, in a 1.0 M LiClO_4_-PC electrolyte. **c** Thickness dependence of transmittance. Neutral (black) and doped (red) state of 150 nm PEDOT film deposited on grid-patterned gold/DSP silicon at the wavelength of 10 μm.(inset) Thickness dependence of transmittance contrast and transmittance contrast ratio. **d** Thermal images of grid-patterned gold/AR-coated germanium and neutral and doped state of 150 nm PEDOT film on grid-patterned gold/AR-coated germanium

PEDOT appears opaque in the oxidized state and transparent in the reduced state. Fig. 1 (a) presents the transmittance (T) spectra from 2.5 to 25 μm of 150 nm-thick PEDOT film deposited on grid-patterned gold/DSP silicon obtained at every 0.1 V between +0.8 V and −0.8 V *versus* the Ag/AgCl (KCl_sat_) reference electrode. The resulting spectra clearly show changes in transmittance as a consequence of the reduction and oxidation of the PEDOT film. As the electrode potential shifts from positive to negative voltage, the transmittance of infrared from 2.5 μm to 25 μm increases resulting from the progressive reduction of PEDOT from its doped state to its neutral state.

From the cyclic voltammograms of the PEDOT films shown in Fig. 1 (b), the forward scan of the PEDOT films leads to the doped state, and the backward scan of the PEDOT films leads to the neutral state, which is transparent in the IR region. An increase in the anodic and cathodic current of thicker film either pumps more ions into the film or extracts more of them from the film. The increased reaction activity of the PEDOT films eventually enhances the transmittance contrast ratio ((T_neutral_-T_doped_)/T_neutral_) in thicker film. Fig. 1 (c) shows the transmittance of the neutral and the doped states of the PEDOT film deposited on grid-patterned gold/DSP silicon with variations in thickness at a wavelength of 10 μm. When the thickness of the PEDOT film increased, the transmittance contrast ratio increased. However, the transmittance contrast (T_neutral_-T_doped_) does not show a consistent tendency as seen in the inset of Fig. 1 (c). As the thickness of the PEDOT film increases, the transmittance contrast increases rapidly, it is maintained at more than 0.16 between 70 nm and 150 nm, and decreases. Since both the transmittance contrast and the transmittance contrast ratio are important for transmissive electrochromic devices, we chose a 150 nm-thick PEDOT film for the optimized device.

Figure 1 (d) shows thermal images of i) AR-coated germanium and 150 nm-thick PEDOT, ii) doped and iii) neutral, over grid-patterned gold/AR germanium on a heated KAIST logo. The temperature of the doped state and the neutral state of PEDOT film was 32.2 °C and 31.6 °C, while the background was 30.6 °C, and the temperature of the substrate without PEDOT film was 34.2 °C. As shown in the FT-IR transmittance spectra result (Fig. 1 (a)), thermal images also reveal that the neutral state of PEDOT film is more transparent than its doped state.

To minimize transmittance loss while maintaining the electrical conductivity, we adopted four different geometries of gold grid whose linewidths were 5 μm and 20 μm and line spaces were 200 μm and 500 μm. As expected, Additional file 1: Figure S1 (a), shows the linear correlation between the planar coverage of gold and transparency. The small deviation could be due to contamination of carbon residues and patterning error.

Transmittance has a positive correlation with open area ratio; sheet resistance also has a positive correlation with open area ratio. To find out the effect of the sheet resistance, a double potential step chronoamperometric experiment was performed (E_1_ = +1.0 V, E_2_ = −1.0 V *versus* Ag/AgCl(KCl_sat_) reference electrode; t_1_ = t_2_ = 30 s). The current versus time profile is shown in Additional file 1: Figure S1 (b). The sheet resistance of the gold grid was lower, and the electrochemical insertion and desertion of ions into PEDOT were saturated faster.

**Fig. 2:**
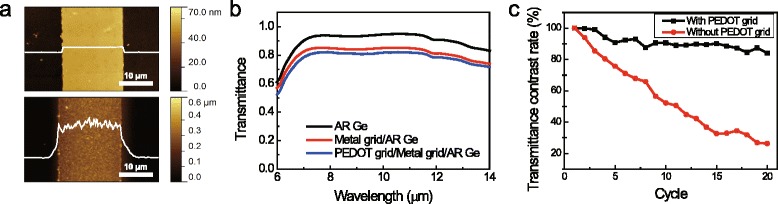
**a** AFM images of (up) grid-patterned gold /substrate and (down) grid-patterned PEDOT/grid-patterned gold/substrate. The images suggest that PEDOT grid only deposited on gold grid. **b** Transmittance spectra of AR-coated germanium, gold grid/AR-coated germanium, and PEDOT grid/gold grid/AR-coated germanium. **c** Transmittance contrast rate of the devices with and without grid-patterned PEDOT. Applied potential ±2.5 V during 20 s

The grid-patterned gold is transparent and electrically conductive; however, it has low durability due to the metal decomposition during doping and undoping of PEDOT as shown in Fig. 2 (c). Therefore, we introduced grid-patterned PEDOT as an ion storage layer. The grid-patterned PEDOT was electrochemically polymerized on pre-patterned grid gold where electrochemical polymerization occurred. Fig. 2 (a) and Additional file 2: Figure S2 show AFM and SEM images of the grid-patterned gold before electrochemical polymerization and after electrochemical polymerization. Because the PEDOT was only polymerized on the grid-patterned gold part, the PEDOT on the grid-patterned area does not seem to have had much effect on the transmittance of the device as shown in Fig. 2 (b). After grid-patterned PEDOT deposition, the device showed stable cyclability, and the transmittance contrast rate was saturated at 80 %. All things considered, including open area ratio, which determines the thickness of the grid-patterned PEDOT, transmittance, conductivity, and response time, we adopted grid-patterned gold with line widths of 20 μm and line spaces of 500 μm as an electrode.

To maximize the transmittance contrast of the device, it is important to use a transparent electrolyte. Additional file 3: Figure S3 shows the transmittance of silicon/electrolyte/silicon the thickness of the electrolyte was varied, which was done using a spacer (2, 10, and 250 μm). When the thickness of the electrolyte was 250 μm, less than 5 % of IR light (λ = 10 μm) could penetrate; however, 60 % of IR light penetrated when the thickness was lower than 10 μm. The thickness of the electrolyte was fixed at 10 μm to maximize the transmittance while preventing electrical short circuits that can be caused when the thickness of the electrolyte is less than 10 μm.

**Fig. 3:**
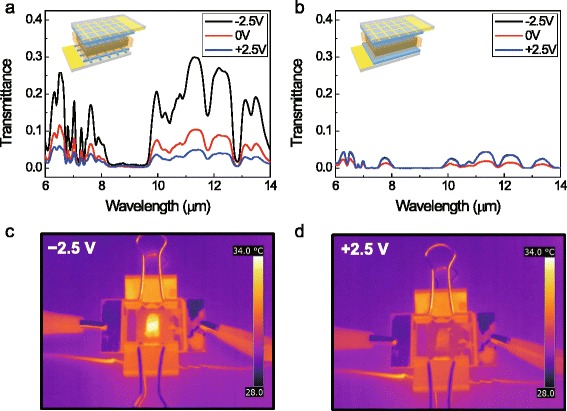
Transmittance spectra of neutral state (−2.5 V), slightly doped state (0 V), and doped state (+2.5 V) of **a** the asymmetric device consisting of 150 nm PEDOT film and grid-patterned PEDOT and **b** the symmetric device consisting of 150 nm PEDOT films. Thermal images of **c** neutral and **d** doped state of device in front of heated bulb

We made two types of devices; one had an asymmetrical configuration, and the other had a symmetrical configuration. The asymmetrical device consisted of 150 nm-thick PEDOT film as a working electrode and grid-patterned PEDOT as a counter electrode; the symmetrical device consisted of two 150 nm-thick PEDOT films as a working electrode and a counter electrode. The spectra were measured using FT-IR at the voltages of +2.5, 0, and −2.5 V. Fig. 3 (a) shows the transmittance spectra of the neutral state (−2.5 V), the slightly doped state (0 V), and the doped state (+2.5 V) of the asymmetrical device, and Fig. 3 (b) shows the transmittance spectra of the opaque state (0 V) and the transparent state (+2.5 V and −2.5 V) of the symmetrical device. The transmittance contrast and transmittance contrast ratio of the symmetrical device were poorer than those of the asymmetrical device as shown in Additional file 4: Figure S4. The PEDOT film of the asymmetrical device can affect the device transmittance without any disturbance of the counter electrode. However, in the case of the symmetrical device, when one side is doped/neutral, the other side is neutral/doped, and the overall transmittance does not change.

**Fig. 4:**
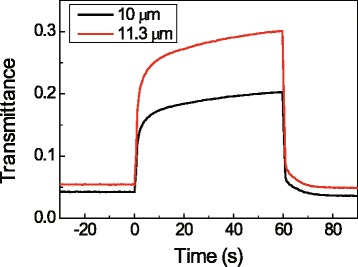
Transmittance spectra of 10 μm and 11.3 μm under alternating potential steps (±2.5 V). Negative potential was applied at 0 s and positive potential was applied after 60 s

Figures 3 (c) and (d) show thermal images of the neutral state (−2.5 V) and the doped state (+2.5 V), respectively, of the IR electrochromic device above a heated bulb. The temperature of the bulb is 40 °C. When the device was in the doped state, it blocked most of the IR light, so the heat source was measured, and its temperature was found to be 31.1 °C, which differs by only 0.9 °C compared to the temperature of the background. On the other hand, the bulb temperature was 34.0 °C when the device was in the neutral state, which makes it possible to clearly distinguish the heat source from the background using the thermal image. Supporting movie file (Additional file 5) also shows the transmittance change of the device. To measure the kinetics of the device, the transmittance changes of the device, shown in Fig. 4, were monitored using real-time FT-IR at the same time as a square wave voltage (−2.5 V to +2.5 V) was applied to the device. The doping and undoping times (to 90 % of equilibrium value) were 1.4 s and 23 s, respectively. The slower switching time of the undoping reaction compared to that of the doping reaction is consistent with those observed in other reports [28–31].

## Conclusions

In conclusion, we studied the transmittance control of PEDOT conducting polymer in the IR range and presented a facile fabrication method of an IR transmissive electrochromic device (transmittance contrast ratio 83 %). We analyzed the transmittance change versus thickness of PEDOT film and determined the appropriate thickness for the transmissive electrochromic device. Grid-patterned PEDOT as an ion storage layer can be easily prepared with electrochemical polymerization on the grid-patterned gold/substrate which is transparent in the IR region. Moreover, the electrolyte was optimized in consideration of both the transmittance and electrical short problems. Finally, the device was analyzed using both FT-IR and an infrared camera. The device exhibited a switching time for bleaching of about 23 s and a switching time of darkening of about 1.4 s. This study has demonstrated transmittance control and a facile fabrication method of an electrochromic device in the IR range, which is meaningful for the field of electrochromic devices and has potential for use in military camouflage.

## Additional files

Additional file 1: Figure S1. (a) Open area ratio versus transmittance and conductivity. (b) Oxidative and reductive current during switching from 1 V to −1 V for PEDOT/grid-patterned gold.
Additional file 2: Figure S2. SEM images of (a) grid-patterned gold/substrate and (b) grid-patterned PEDOT/grid-patterned gold/substrate.
Additional file 3: Figure S3. Transmittance spectra with variation of thickness of electrolyte.
Additional file 4: Figure S3. Transmittance contrast and transmittance contrast ratio of (a) asymmetric device and (b) symmetric device.
Additional file 5:
**Supporting Movie.**

## Abbreviations

PEDOT: Poly(3,4-ethylenedioxythiophene)
MWIR: Mid-wavelength infrared
LWIR: Long-wavelength infrared
PANI: polyaniline
CSA: Camphor sulfonic acidv
ITO: Indium tin oxide
DSP: Double-side polished
EDOT: 3,4-ethylenedioxythiophene
AFM: Atomic force microscope
FT-IR: Fourier transform infrared spectroscopy
T: Transmittance
