# Supplementary figures and images for: Patternable PEDOT nanofilms with grid electrodes for transparent electrochromic devices targeting thermal camouflage
Source: Nano Converg. 2015 Oct 1;2(1):19. doi: 10.1186/s40580-015-0051-9 (PMC5270994; doi:10.1186/s40580-015-0051-9)

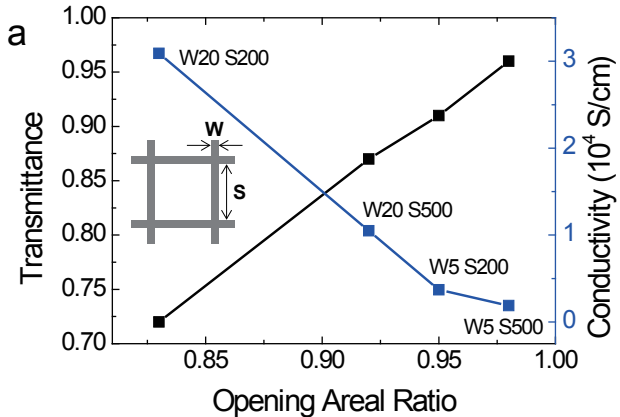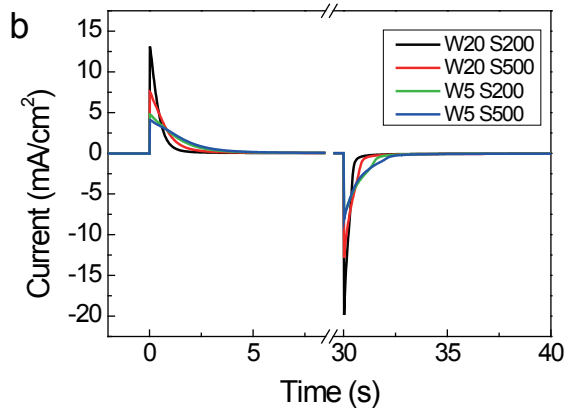

Supplement: Additional file 1: Figure S1. — (a) Open area ratio versus transmittance and conductivity. (b) Oxidative and reductive current during switching from 1 V to −1 V for PEDOT/grid-patterned gold. [file 40580_2015_51_MOESM1_ESM.pdf]

a

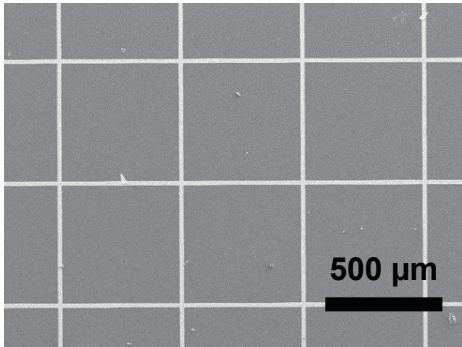

b

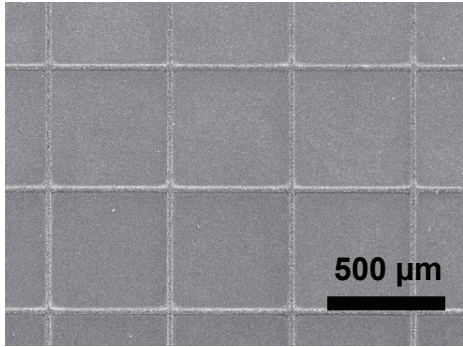

Supplement: Additional file 2: Figure S2. — SEM images of (a) grid-patterned gold/substrate and (b) grid-patterned PEDOT/grid-patterned gold/substrate. [file 40580_2015_51_MOESM2_ESM.pdf]

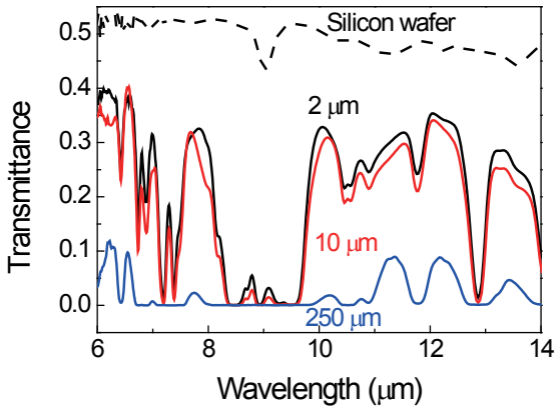

Supplement: Additional file 3: Figure S3. — Transmittance spectra with variation of thickness of electrolyte. [file 40580_2015_51_MOESM3_ESM.pdf]

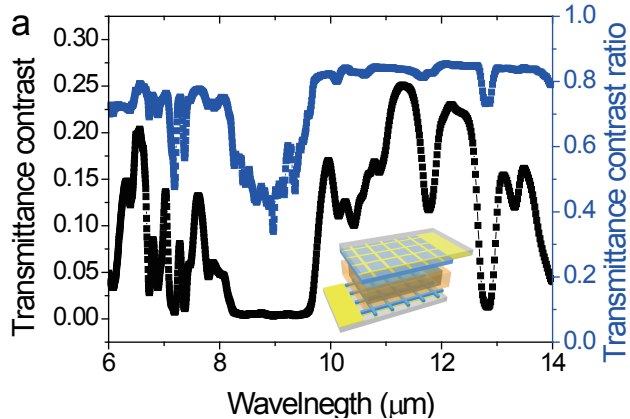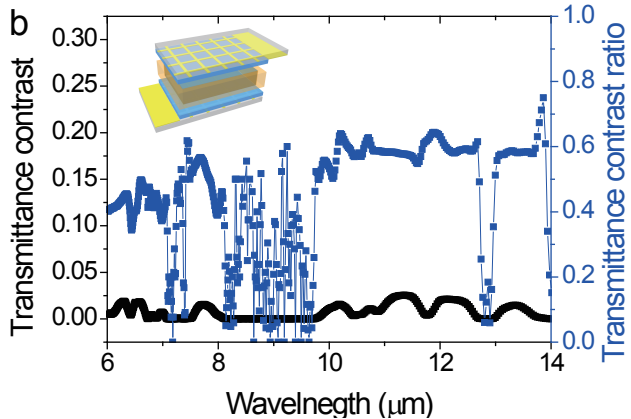

Supplement: Additional file 4: Figure S3. — Transmittance contrast and transmittance contrast ratio of (a) asymmetric device and (b) symmetric device. [file 40580_2015_51_MOESM4_ESM.pdf]
